# Dietary unsaturated fatty acids affect the mammary gland integrity and health in lactating dairy cows

**DOI:** 10.1186/1753-6561-5-S4-S35

**Published:** 2011-06-03

**Authors:** Núria Mach, Jürgen van Baal, Leo Kruijt, Antoon Jacobs, Mari Smits

**Affiliations:** 1 Animal Breeding and Genomics Centre, Wageningen UR Livestock Research, P.O. Box 65, 8200 AB Lelystad, The Netherlands; 2 Animal Nutrition Group, Wageningen University, P.O. Box 338, 6700 AH Wageningen, The Netherlands

## Abstract

**Background:**

Information about the effects of unsaturated fatty acids (UFA) supplementation on the health and integrity of the mammary gland in lactating dairy cows is lacking. Therefore, the aim of this study was to determine the effects of unprotected dietary UFA on the global expression pattern of genes in the mammary gland tissue of grazing dairy cows, and to translate this information into relevant biological knowledge.

**Methods:**

Twenty-eight Holstein-Friesian dairy cows were randomly assigned to 4 different concentrated UFA-sources for 23 days after which all cows were switched to a non-UFA-supplemented concentrate for an additional 28 days. On the last day of both periods, mammary gland biopsies were taken to study genome-wide differences in gene expression on Bovine Genome Arrays.

**Results:**

Supplementation with UFA reduced the concentration of short chain fatty acids (FA), C16 FA and saturated FA in the milk, whereas that of *trans-*FA increased. One major finding was that canonical pathways associated with remodelling and immune functions of the mammary gland were predominantly down-regulated during UFA supplementation and negatively correlated with the concentration of milk *trans*-FA.

**Conclusions:**

Supplementing grazing dairy cows with unprotected dietary UFA can affect the remodelling and immune functions of the mammary gland with potential consequences for its integrity and health, as well as milk quality.

## Background

Because of the potential health benefits of unsaturated fatty acids (UFA), there is considerable interest in increasing their concentration levels in milk. The effect of supplementing dietary UFA on the resulting milk fatty acid (FA) composition and expression of several genes involved in the mammary gland lipid metabolism has recently been studied in dairy cows [1,2]. However, information about the effects of UFA supplementation on genome-wide expression of genes in the mammary gland tissue of dairy cows, as well as their relationship with the resultant milk FA composition, is lacking. Identifying these effects is expected to contribute to our understanding of milk fatty acid metabolism in the mammary gland tissue, and to enhance opportunities to improve milk fat composition through nutrition. Therefore, the aim of this study was to determine the effects of unprotected dietary UFA on the global expression pattern of genes in the mammary gland tissue of grazing dairy cows, to translate this into more biological knowledge, and to correlate gene expression patterns to the resulting milk fatty acid composition.

## Methods

Twenty-eight Holstein-Friesian dairy cows were randomly assigned to 4 concentrated UFA-sources based on either unprotected rapeseed oil, soybean oil, linseed oil, or a proportional mix of these plants oils for 23 days (Period I), after which all cows were switched to a non-UFA-supplemented concentrate for an additional 28 days (Period II). On the last day of both periods, mammary gland biopsies were taken to study genome-wide differences in gene expression using Affymetrix GeneChip® Bovine Genome Arrays. In addition, milk samples were taken and stored at -20°C until analysis for FA composition by gas chromatography. Milk FA composition was analyzed using a mixed-effects ANOVA (SAS Inst. Inc. Cary, NC, release 9.1). The model included UFA-sources, experimental periods, and the interaction between UFA-sources and the experimental period, as fixed effects, and cow within pen as a random effect. The same model was performed to analyse gene expressions (MAANOVA package of R, release 1.16). The list of differentially expressed genes was generated using a False Discovery Rate (FDR) < 5% together with an absolute fold-change (FC) threshold of 1.3. Additionally, the Ingenuity Pathways Analysis (IPA; ver. 5.5., Ingenuity Systems, Redwood City, CA) was applied to identify the relevant genes associated with molecular and cellular functions, canonical pathways and biological functions, as well as the biological interaction networks among significant genes. As a means to potentially identify the association between differentially expressed genes in the mammary gland tissue affected by unprotected dietary UFA, and the resulting milk FA composition, the relationship between milk FA groups and mRNA expression of affected genes was calculated using the mixOmics package of R (release 2.7). The significance of the correlations described was tested by linear model using limma package of R.

## Results and discussion

Supplementation of dietary UFA decreased (*P* < 0.01) the concentration of short chain fatty acids (SCFA), 16-carbon FA (C16), and saturated fatty acids (SFA), and increased the concentration of conjugated linoleic acid (CLA) isomers (Table 1), suggesting that supplementing dairy cows with dietary UFA may help to improve the health and nutrition quality aspects of dairy milk. However, the omega-3 (n-3) and omega-6 (n-6) FA concentration decreased, and the concentration of *trans-* FA in milk increased with UFA supplementation (Table 1). Applying a statistical cut-off of FDR <0.05 together with an absolute FC threshold of 1.3, we identified a total of 972 genes as being differentially expressed in the mammary gland tissue when supplementing dairy cows with UFA compared with control diet. The expression of genes in mammary gland was not significantly affected by the different dietary unprotected UFA-sources. The genes affected by UFA supplementation were involved in molecule transport, lipid and protein metabolism, cell growth and proliferation, remodelling, and defense, inflammatory and immune response. A specific examination of the canonical pathways involved in remodelling and immune system using IPA revealed that UFA supplementation down-regulated a total of 78 genes associated with cell signalling pathways, including mTOR, JAK/Stat, prolactin and integrin signalling, as well as cellular and humoral immune responses, pathogen-induced signalling and cellular stress and injury. Ranking of the affected biological networks identified the *P53* protein, a key transcription factor that controls mammogenesis in ruminants, as the major traffic controller.

**Table 1:** Milk fatty acid concentration (mg/L) when comparing dairy cows fed with unsaturated fatty acids (UFA) enriched-diet relative to the same cows fed control diet

	Diet supplementation			
			
	Non-UFA	UFA	SEM	*P*- value^1^
	
Item^2^				UFAL
	
Short Chain Fatty Acids, mg/L	11,343	8,035	418.5	<0.001
^3^16C, mg/L	13,200	9,780	416.7	<.0001
Long-Chain Fatty Acids, mg/L	15,282	15,412	467.9	0.84
Unsaturated Fatty Acids, mg/L	12,023	12,397	349.6	0.45
Saturated Fatty Acids, mg/L	28,393	21,160	938.6	<.0001
Polyunsaturated Fatty Acids, mg/L	1,393	1,388	43.1	0.93
^4^n-3 Fatty Acids, mg/L	269.3	201.7	9.28	<.0001
^5^n-6 Fatty Acids, mg/L	721.1	652.46	20.01	0.01
*Trans*-octadecenoic Fatty Acids, mg/L	1,798	2,900	72.5	<0.001
*cis*-9, *trans*-11-CLA, mg/L	263.9	368.8	18.80	0.002
*trans*-10,*cis*-12-CLA^f^, mg/L	3.56	6.4	0.46	<0.0001

^1^*P*- value = effect of unsaturated fatty acids enriched-diet supplementation

^2^Included n= 28 cows

^3^16C = sum of 16:0 and 16:1,*cis*-9

^4^Sum of n-3 fatty acids (18:3n-3, 20:3n-3, 20:5n-3, and 22:5n-3)

^5^Sum of n-6 fatty acids (18:2n-6, 18:3n-6, 20:2n-6, 20:3n-6, 20:4n-6, and 22:2n-6)

**Figure 1:**
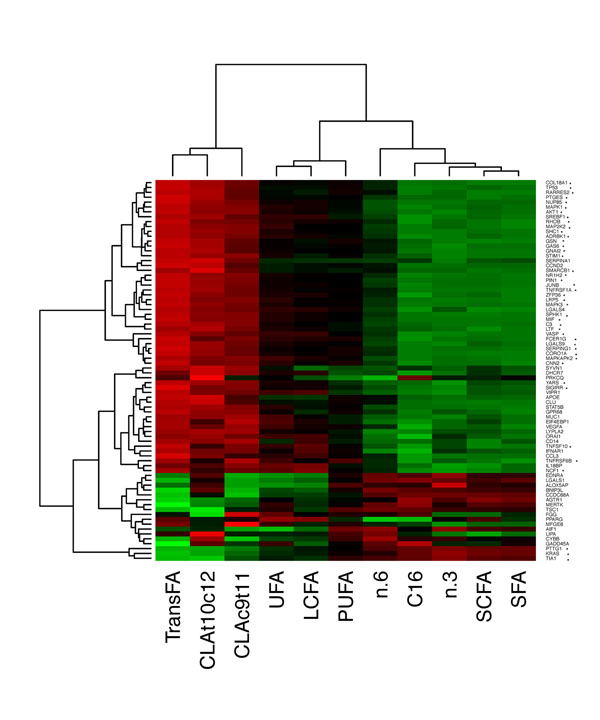
**Milk fatty acids correlated to remodelling and immune response related-genes**. Heat map of the correlations between the 78 significantly expressed genes involved in remodelling and immune response-related genes when comparing dairy cows supplemented with unsaturated fatty acid relative to the same cows fed a control diet, and the milk fatty acids content. The 78 immune function-related genes were identified by Ingenuity Pathways Analysis and presented a Fold Change (FC) ≥ 1.3 and a false discovery rate (FDR) *q*-values < 0.05. The horizontal rows of the map represent genes, whereas the columns represent the different groups of fatty acids. Each pixel represents the correlation value between each gene and group of fatty acids: the colours depict the coefficient of correlation from green (large negative) to red (large positive). * indicates gene signatures that presented a significant correlation with the FA in milk

Little is known about remodelling, defense, and inflammatory and immune-related genes in response to dietary UFA supplementation in dairy cows. However, results presented here suggest that dietary UFA supplementation may affect mammary gland tissue integrity and cell adhesion, as well as immune functions, and thus may modify the susceptibility to mastitis in lactating cows and the resulting quality of milk. To determine whether the expression of these genes correlated with specific milk FA, we then studied the association between the set of 78-remodelling and immune response genes and different groups of FA. As depicted in Figure 1, we detected that the characterized FA groups could be divided into two main clusters, each with a distinctive correlation value to remodelling and immune response genes. A novel finding was that the first main cluster included the milk *trans*-FA and CLA isomers, and the second cluster the other groups of FA (SFA, C16, SCFA, n-3, n-6, and LCFA, UFA, and polyunsaturated FA (PUFA)). Furthermore, whereas *trans*-FA and CLA isomers negatively correlated with remodelling and immune response gene expressions (Figure 1), the other groups of FA showed a positive correlation with the level of remodelling and immune response gene expressions. This results are supported by the discovery that t10,c12 CLA isomer modulated the regulation of gene expression associated with immune system [3]. However, evidence for the effects of *trans*-FA on remodelling and immune-gene expression in the mammary gland of dairy cows is currently lacking. Remarkably, the regulation of gene expression by these FAs seems to be due to changes in the expression of different cell signalling and transcription factors related to a wide range of metabolic and developmental functions, among them, *P53*, *ZFP36* (zinc finger protein 36) and *SREBP1* (sterol regulatory element binding protein 1; Figure 1). *ZFP36* has anti-inflammatory activity by binding to and destabilizing pro-inflammatory mRNAs, such as tumor necrosis factor-alpha (*TNF-*α) mRNA [4]. Further, *P53* is a transcription factor regulating DNA repair, apoptosis, cell cycle arrest, and senescence [5], and *SREBP1* is a key regulator of intracellular lipid homeostasis. Notably, recent results suggest that the immune system, through Lymphotoxin-beta receptor (LTBR) signalling, directly influences the enzymatic regulation of lipid homeostasis and thus the *SREBP1* activation [6]. Knowledge of the mechanisms by which FA control specific gene expression may provide insight into the development of new nutrition strategies for a better management of milk quality and mammary gland health status.

## Conclusions

The results of this study show that supplementing grazing dairy cows with different unprotected unsaturated fatty acids reduced the concentration of short chain fatty acids, C16 and saturated fatty acids in the milk, whereas that of *trans-*fatty acids increased. Dietary unsaturated fatty acid supplementation affected 78 remodelling and immune response-related genes, suggesting an effect on mammary gland tissue integrity and health. A novel finding was that the milk *trans-*FA presented a significant and negative correlation profile to these genes, therefore it is tempting to speculate on an active role of this FA-group in the regulation of mammary gland gene expression associated with immune system and remodelling components. Further functional knowledge by which fatty acids control these genes will be of great importance in understanding the resulting mammary gland health and integrity, as well as the milk composition and quality.

## Competing interests

The author(s) declare that they have no competing interests.
